# Analysis of off-label drug use and its influencing factors in pediatric patients undergoing otolaryngology, head, and neck surgery

**DOI:** 10.3389/fphar.2025.1553221

**Published:** 2025-05-30

**Authors:** Zichao Ji, Xin Wang, Jiawei Wang

**Affiliations:** Department of Pharmacy, Beijing Tongren Hospital, Capital Medical University, Beijing, China

**Keywords:** off-label, pediatric patients, prescription, outpatient, otolaryngology, head and neck surgery, influencing factor

## Abstract

**Background:**

To date, there are limited data regarding off-label drug use in pediatric patients undergoing otolaryngology, head, and neck surgery. This study aimed to investigate the prevalence and patterns of off-label drug use among outpatients under 18 years of age in the Otolaryngology, Head, and Neck Surgery department, and to analyze the factors influencing such usage.

**Methods:**

A retrospective collection of data was conducted based on outpatient prescriptions for patients under 18 years of age who were visited from November 2023 to October 2024. Clinical pharmacists collected and analyzed off-label drug use. Univariate and multivariate logistic regression were employed to analyze the factors associated with off-label drug use.

**Results:**

In total, 22,487 prescriptions were included, of which 2,269 were off-label, resulting in an incidence rate of off-label drug use of 10.09%. Among 2,269 off-label prescriptions, the most common type was off-label population use (2,090 instances), accounting for 92.11%. The 2,269 off-label prescriptions encompassed 2,359 records of off-label drug use. Limonene and Pinene enteric capsules had the highest frequency of off-label use, with 1,523 records, representing 64.56% of all off-label uses. Multivariate logistic regression analysis showed that patients’ age (OR = 1.341, 95% CI: 1.324 ∼ 1.359, p = 0.000), number of prescribed drugs (OR = 1.597, 95% CI: 1.528 ∼ 1.671, P < 0.001), and physician title were the factors associated with off-label drug use. Attending physicians (OR = 0.555, 95%CI: 0.458 ∼ 0.673, p < 0.001), deputy chief physicians (OR = 0.770, 95%CI: 0.637 ∼ 0.930, p = 0.007), and chief physicians (OR = 0.419, 95%CI: 0.347 ∼ 0.507, p < 0.001) were less likely to have off-label drug use compared to resident physicians.

**Conclusion:**

These findings indicate that pediatric patients who are older and receive a greater variety of medications are more likely to use drugs off-label. In addition, resident physicians exhibit a higher susceptibility for off-label drug use compared to attending physicians, deputy chief physicians, and chief physicians.

## 1 Introduction

Off-label drug use (OLDU), also referred to as off-label use of drugs and unregistered use of drugs, pertains to the use of medications that fall outside the approved indications, dosages, treatment durations, administration routes, or patient populations specified in the instructions of the drug regulatory department (Clinical Pharmacology Group of Pediatrics Branch of Chinese Medical Association, 2016). This type of practice is widely prevalent both domestically and internationally in different medical fields. Studies conducted in the United States have revealed that approximately 21% of commonly prescribed medications in general outpatient clinics are employed off-label (Radley et al., 2006). Moulis et al. indicated that off-label drugs are prescribed for 7%–51.7% of children in outpatient settings (Moulis et al., 2018). A study published in 2024, which assessed the global rates of off-label and unapproved prescriptions among hospitalized children, reported that 46% of pediatric patients worldwide receive off-label prescriptions during their hospital stay. Subgroup analysis showed that the highest proportion (54%) can be seen among Australian pediatric patients, followed by Asian (52%), South American (45%), European (42%), and North American (38%) pediatric patients (Yuan et al., 2024). However, there are limited data on off-label drug use among pediatric patients in China. Outpatient data from a tertiary-level A children’s hospital in China indicated that the incidence rate of off-label drug use can reach up to 53% in pediatric outpatient prescriptions (Guo and Wang, 2014).

The main reason behind off-label drug use is the delay in updating drug instructions by the National Medical Products Administration compared to advancements in clinical diagnosis and treatment technology. The approval of new drugs often relies on limited clinical data, while numerous discoveries and experiences emerge during clinical practice. Due to the complex process of updating drug instructions, pharmaceutical companies need significant time and resources to accomplish registration-compliant clinical research, which can prolong the updating process of drug instructions compared to the progress made in clinical practice. Off-label drug use is an inevitable reality. Herein, a United Kingdom study investigated the association of off-label drug use with adverse drug reactions (ADR) among hospitalized children and found that compared to authorized drugs, off-label and unlicensed drugs are associated with a higher risk of ADR (Bellis et al., 2014).

Therefore, the current situation and safety of off-label drug use in pediatric patients have always been a research focus. Through a systematic review of literature from PubMed, Embase, CNKI, and Wanfang Databases over the past decade, we identified a substantial gap in research regarding the off-label use of drugs among patients younger than 18 years of age who undergo otolaryngology, head, and neck surgery. Notably, the Department of Otolaryngology at Beijing Tongren Hospital ranked second in the 2023 China Hospital Specialty Reputation Ranking, making it a leading institution that attracts patients nationwide.

This study aimed to present the current status of off-label drug use among patients under 18 years of age at the Department of Otolaryngology, Head, and Neck Surgery. Additionally, this study aimed to analyze the factors leading to off-label drug use in this population.

## 2 Materials and methods

### 2.1 Setting and study design

A retrospective collection of data was conducted at Beijing Tongren Hospital. Established in 1886, this hospital is a tertiary hospital renowned for its national key disciplines in ophthalmology and otolaryngology with a total of 1,611 beds. It annually provides medical care to more than 2.7 million outpatients and those who need emergency care. The Puhua Hecheng Rational Drug Use System is currently one of the most extensively utilized software for rational drug use in China. It provides medical institutions with real-time intelligent prescription reviews, post-prescription assessments, and patient medication guidance, thereby establishing a comprehensive professional safeguard system for pharmacotherapy. In this study, we employed this system to extract outpatient prescriptions for patients younger than 18 years of age who visited the Department of Otorhinolaryngology and Head and Neck Surgery from November 2023 to October 2024. The prescription management regulations in our country suggest that each prescription should not include more than five types of medications. In this study, the drug and its solvent were documented as one medication. The following types of prescriptions were excluded: (1) inaccurate or incomplete prescriptions; (2) traditional Chinese medicine or hospital preparations; (3) non-pharmaceutical prescriptions (consumables, etc.); and (4) follow-up medication documentation. The study was conducted using the principles outlined in the Declaration of Helsinki, and the study protocol was approved by the Ethics Committee of Beijing Tongren Hospital. Due to the retrospective nature of this study, patients’ consent was not necessary for inclusion.

### 2.2 Data collection and collation

Microsoft Excel 2021(Version 2504 Build 16.0.18730.20122) was employed to collect the following patients’ data and medication data obtained from a single visit through the Puhua Hecheng Rational Drug Use System: patient identification number, age, gender, prescription number, prescription date, department, diagnosis, drug name, single dose, frequency of administration, route of administration, and physician’s professional title. The diagnostic category for each patient was assigned using the International Classification of Diseases 10th Edition (ICD-10).

Off-label drug use was determined based on the product description. Each prescription was reviewed and classified based on three aspects: indication, appropriate target population (no medication information for those younger than 18 years of age and being outside the age range), and dosage (single dose, frequency of administration, and route). The drug was recorded if: 1) it lacked pediatric information; 2) the age range fell outside the specified range; 3) a single dose exceeded the recommended dosage; or 4) the drug was administered more frequently than indicated. Multiple off-label uses could be recorded for each medication message. However, if a drug was used for non-target populations, we could not evaluate whether its single dose and frequency of administration were off-label.

Ji ZC and Wang X, two senior clinical pharmacists, independently evaluated prescriptions and resolved inconsistent judgments through discussion or consultation with experts. The intraclass correlation coefficient (ICC), a two-way mixed model, was employed to assess consistency between the two assessors. As suggested by Koo and Li, (2016), a score of more than 0.75 indicates good/excellent agreement and is acceptable for further analyses.

The patients were divided into two groups based on the presence or absence of off-label drug use. According to the age classification of the European Medicines Agency (EMA), the age of patients was divided into four groups: newborns (0–27 days), infants (28 days–23 months), children (2–11 years), and adolescents (12–18 years). Preterm infants were excluded, and only those with a corrected age of 40 weeks or older were included in this study. Medical data and discrepancies in off-label drug use were compared between the group with off-label drug use and the group without such use. Additionally, the factors associated with off-label drug use among patients younger than 18 years of age in the departments of otorhinolaryngology, head, and neck surgery were systematically analyzed.

### 2.3 Statistical analysis

Data were recorded and organized using Microsoft Excel (Version 2504 Build 16.0.18730.20122). IBM SPSS Statistics (27.0.1) was employed for data processing and analysis. Qualitative data are presented as frequencies and percentages (%), whereas quantitative data are presented as mean ± standard deviation. The t-test was employed to analyze measurement data, while the chi-squared test was employed to analyze count data. First, univariate logistic regression analysis was conducted, and P < 0.1 was considered statistically significant. Collinearity was assessed on variables with P < 0.1 in univariate analysis. Variables with a variance inflation factor (VIF) > 5 were excluded from the analysis. Subsequently, multivariate binary logistic regression analysis was conducted to identify independent risk factors associated with off-label drug use. Excessive drug use was defined as the dependent variable. Gender and physician rank were regarded as independent variables. P < 0.05 was considered significant in multivariate analysis.

## 3 Results

### 3.1 General situation

In total, 60,328 medication records were obtained, of which 430 were excluded due to inaccuracies or inconsistencies. Among the remaining records, 12,880 belonged to traditional Chinese medicine, 3,283 were hospital preparations, and 320 were non-pharmaceutical items. Additionally, we found 7,669 return visit records. After careful analysis and refinement, 35,746 medication records were finally included in this study. This comprehensive dataset comprised 22,487 prescriptions and 20,757 patients. Among patients, 63.43% were males. Children accounted for the largest number of patients, accounting for 77.12% of the total participants, and the chief physician patients constituted the largest number of patients, accounting for 36.50% of the total population. The prescriptions and basic characteristics of the included patients are shown in Table 1.

**TABLE 1:** Prescription details and patient demographics.

Parameter	N	%
	20,757	100
Gender		
Male	13,167	63.43
Female	7,590	36.57
Age		
Newborns (0–27 days)	0	0
Infants (28–23 months)	83	0.40
Children (2–11 years)	16,007	77.12
Adolescents (12–18 years)	4,667	22.48
The expertise of the prescribing physician		
Resident physician	1,672	8.06
Attending physician	6,332	30.51
Deputy chief physician	5,175	24.93
Chief physician	7,578	36.50
The number of prescribed drugs		
1	10,363	49.93
2	7,088	34.15
3	2,498	12.03
4	564	2.72
5	119	0.57
>5	125	0.60

N, number of patients.

### 3.2 Overview of off-label drug use

The analysis encompassed 35,746 drug use records, of which 2,359 were classified as off-label use, resulting in an incidence rate of 6.60%. Among the examined drug records, there were 22,487 prescriptions involving 2,269 instances of off-label prescribing with an incidence rate of 10.09%. Of all patients (20,757), there were 2,242 cases with off-label drug use, with a prevalence of 10.80%. Among the included medication records (35,746), there were 130 drugs, all of which were in the formulary of our hospital. Among the 130 drugs, 32 were used off-label, representing 24.62% of the total drug use.

### 3.3 Different types of off-label drug use

Among the types of off-label drug use for outpatient patients under 18 years old in the Department of Otolaryngology, Head and Neck Surgery, the incidence of using drugs beyond the recommended population in the drug instructions is the highest. The analysis encompassed 35,746 drug use records, of which 1,404 were classified as off-label use based on the target population, resulting in an incidence rate of 3.93%. Among the examined drug records, there were 22,487 prescriptions involving 1,376 instances of off-label prescribing with an incidence rate of 6.12%. Further details can be found in Table 2.

**TABLE 2:** Incidence of different types of off-label drug use.

Types of off-label drug use	Medication record (N = 35,746)		Prescription (N = 22,487)	
	Off-label drug use (N)	Incidence rate (%)	Off-label drug use (N)	Incidence rate (%)
Off-label use based on the target population	1,404	3.93	1,376	6.12
Off-label use based on the target population and indication	714	2.00	714	3.18
Off-label use based on the target indication	168	0.47	166	0.74
Off-label use based on the route of administration	73	0.20	69	0.31

### 3.4 Drug off-label analysis

Among 35,746 drug use records, 2,359 were off-label drug use records. Notably, off-label drug use records for Limonene and Pinene enteric capsules ranked first with 1,523 records, representing 64.56% of all off-label drug use records. The 1,523 records were primarily attributed to the off-label population use, with 700 off-label indications specifically for the treatment of otitis media, secretory otitis media, chronic rhinitis, and allergic rhinitis. Bencycloquidium bromide nasal spray (13.73%) had the second highest number of off-label use records, followed by azdrostine hydrochloride nasal spray (4.45%). Detailed information about the top 10 drugs in terms of off-label drug use is presented in Table 3.

**TABLE 3:** Off-label drug use records of the top 10 drugs.

Ranking	Generic name	Off-label medication records (N)	Percentage of off-label medication records (%)	Off-label use
1	Limonene and Pinene enteric capsules	1,523	64.56	<18 years old (1,523); otitis media, otitis media with effusion, chronic rhinitis, and allergic rhinitis (700)
2	Bencycloquidium bromide nasal spray	324	13.73	<18 years old
3	Azelastine hydrochloride nasal spray	105	4.45	5 years old and under
4	Mecobalamin tablets	85	3.60	<18 years old (85), hearing loss (8)
5	Montelukast sodium chewable tablets	80	3.39	Adenoid hypertrophy
6	Budesonide suspension for inhalation	50	2.12	Pharyngitis, laryngitis, and laryngeal edema
7	Erythromycin eye ointment	48	2.03	Rhinitis, epistaxis, nasal surgery, ear trauma, and ear surgery for external use
8	Budesonide nasal spray	21	0.89	Adenoid hypertrophy, and otitis media with effusion
9	Levofloxacin hydrochloride ear drops	17	0.72	<3 years old
10	Gentamicin sulfate injection	17	0.72	Nasal irrigation

### 3.5 Univariate analysis of off-label drug use

All factors, including sex, age, quantity of prescribed medications, and prescribing physician’s title, were correlated with off-label drug use (p < 0.1) (Table 4).

**TABLE 4:** Single-factor analysis leading to off-label drug use.

Parameter	Off-label drug use group (N = 2,242)	No off-label drug use group (N = 18,515)	OR(95%CI)	p - value
Gender male [N (%)]	1,471(65.61)	11,696 (63.17)	1.112 (1.014–1.220)	0.023
Age (years)	14 (4)	7 (5)	1.355 (1.337–1.372)	0.000
Quantity of prescribed medications	2 (1)	1 (1)	1.758 (1.688–1.831)	<0.001
Prescribing physician’s title [N (%)]				
Resident physician	197 (8.79)	1,353 (7.31)	1	-
Attending physician	594 (26.49)	5,519 (29.81)	0.739 (0.623–0.878)	<0.001
Deputy chief physician	727 (32.43)	4,695 (25.36)	1.063 (0.898–1.259)	0.474
Chief physician	724 (32.29)	6,948 (37.52)	0.716 (0.605–0.847)	<0.001

OR, odds ratio; CI, confidence interval.

### 3.6 Multifactorial analysis of off-label drug use

The statistically significant variables in the univariate analysis, such as sex, age, the number of prescribed medications, and prescribing physician’s title exhibited collinearity. The VIF values of the four factors were all <5. The presence or absence of off-label use was considered the dependent variable. Four variables, gender, age of patient, number of prescribed drugs, and physician’s title, were used to construct a binary logistic regression model. These variables were treated as independent variables. The results showed that age, number of prescribed drugs, and physician’s title were independent risk factors for off-label drug use (Table 5).

**TABLE 5:** Analysis of the factors affecting off-label drug use among outpatients younger than 18 years of age visiting the Department of Otolaryngology, Head, and Neck Surgery.

Parameters	B	S.E.	Wald χ2	OR	95% CI for OR		p-Value
					Lower	Upper	
Sex (male)	0.007	0.052	0.018	1.007	0.909	1.116	0.894
Age	0.294	0.007	1874.894	1.341	1.324	1.359	0.000
Number of prescribed Medications	0.468	0.023	420.193	1.597	1.528	1.671	<0.001
Resident physician	-	-	-	1	-	-	-
Attending physician	−0.588	0.098	35.936	0.555	0.458	0.673	<0.001
Deputy chief physician	−0.262	0.096	7.377	0.770	0.637	0.930	0.007
Chief physician	−0.869	0.097	80.463	0.419	0.347	0.507	<0.001

B, regression coefficient; S.E., standard error; OR, odds ratio; CI, confidence interval.

## 4 Discussion

As far as we know, this is the first study in China that investigated the status of off-label drug use and the associated factors among subjects less than 18 years of age in the outpatient setting of the Department of Otolaryngology, Head, and Neck Surgery. In this study, 22,487 prescriptions were included in the final analysis, including 2,269 off-label prescriptions, and the incidence of off-label drug use was 10.09%. In total, 130 drugs were involved in 22,487 prescriptions, of which 32 were engaged in off-label use, accounting for 24.62%. These findings are consistent with studies from the United States showing that nearly 21% of commonly prescribed drugs in general outpatient clinics were used off-label (Radley et al., 2006).

The type of off-label use with the highest incidence among the pediatric outpatients of the Otolaryngology, Head, and Neck Surgery Department was off-label population drug use, accounting for 2,090 out of 2,269 off-label prescriptions (92.11%). The part about pediatrics medicine is often expressed in the instruction manual as “not clear,” “not recommended for children,” “this experiment has not been conducted and there is no reliable reference,” “generally not for infants and young children” and so on. Due to ethical considerations, pediatric patients (especially newborns) are not included in drug clinical trials, and pharmaceutical companies cannot effectively evaluate the safety and rationality of drugs for this specific group, resulting in a higher incidence of off-label drug use in pediatrics compared to other populations. The second type of off-label use was off-label indication drug use, which accounted for 880 out of 2,269 off-label drug prescriptions, accounting for 38.78%. A study was conducted based on the 8th edition of the list of essential medicines for children issued by the World Health Organization (WHO) and the list of children’s medications issued by the National Medical Products Administration to encourage research and development and the declaration of children’s drugs. The study assessed 561 drug varieties, excluding the categories that were not listed as drugs in China or some specific categories. Among them, 353 varieties have been listed in China, including four types of drugs related to the field of otolaryngology, accounting for 1.1% (Tu et al., 2024). The shortage of drugs related to the otolaryngology specialty is bound to off-label drug use.

The results showed that the Limonene and Pinene enteric capsules ranked first among the top 10 drugs with off-label use. The indications of this drug are acute and chronic sinusitis, acute and chronic bronchitis, pneumonia, etc.; however, its use for pediatrics remains unclear. Among the children included in this study, 700 were treated with Limonene and Pinene enteric capsules for otitis media, otitis media with effusion, chronic rhinitis, and allergic rhinitis. Therefore, it was used was off-label populations and indications. By searching the literature, we found many reports on the use of Limonene and Pinene enteric capsules in the treatment of otitis media, otitis media with effusion, chronic rhinitis, and allergic rhinitis in pediatric patients in China (Tian et al., 2024; Zhu, 2022; Hu, 2018). Limonene and Pinene enteric capsule is a mucolytic expectorant that can potentiate the clearance and defense mechanism of the mucociliary system, break the vicious cycle of inflammation, improve the secretion of respiratory glands, and prevent mucus accumulation in the middle ear and nasal cavity. The drug also has certain antibacterial and anti-inflammatory properties, which can reduce the degree of mucosal swelling, reduce inflammatory cell infiltration, promote the repair of damaged mucosal tissue, and improve disease outcome.

This study not only reported the current situation of off-label drug use in pediatric outpatients of the Department of Otolaryngology, Head, and Neck Surgery of our hospital but also analyzed the factors associated with off-label drug use. We found that patients’ age, the number of prescribed drugs, and the professional title of physicians were independent risk factors for off-label drug use among pediatric patients of the Department of Otolaryngology, Head, and Neck Surgery. Of 20,757 patients included in this study, 83 were younger than 2 years old, accounting for 0.40% of the included patients. Our hospital, a tertiary hospital with an Otorhinolaryngology, Head, and Neck Surgery Department, is located in Beijing, and patients from all over the country come here. Beijing also has two nationally renowned specialized children’s hospitals. The families of pediatric patients less than 2 years of age more often go to children’s specialized hospitals; therefore, a relatively low proportion of patients are less than 2 years of age in our hospital. Our results indicated that the number of prescribed drugs increases with the age of pediatric patients, leading to a higher incidence of off-label drug use. On the contrary, some studies showed that younger subjects are more likely to use off-label drugs (Langerová et al., 2014; Jaberi et al., 2024). Considering that this study was a single-center study and there are regional differences in off-label drug use, the results should be interpreted with caution. However, our findings suggest that off-label drug use can occur at any age among children.

Physicians’ title was an independent risk factor for off-label drug use. This study showed that attending physicians, associate chief physicians, and chief physicians were less likely to have off-label drug use compared to residents. This finding is inconsistent with a study on off-label drug use among children in Gansu Province, which included all children’s prescriptions in tertiary and secondary public hospitals in Gansu Province and found that senior physicians were more likely to prescribe off-label drugs than junior physicians (Meng et al., 2024). On the one hand, residents have little clinical experience and are less familiar with pharmacological effects, target population, and safety of drugs compared to physicians with middle and senior titles. On the other hand, considering the characteristics of otolaryngology, head, and neck diseases, many patients need drug therapy combined with surgical treatment. Children’s families usually choose physicians with higher qualifications and more experience, particularly middle and senior physicians with more effective treatment plans and choices, and less off-label drug use. A Russian study assessed clinical specialists’ knowledge and experience of off-label drug use and showed that 65% of respondents felt that they did not know enough about off-label prescriptions. Nearly 75% of respondents found it useful to have information about the risks and benefits of off-label prescription in clinical practice (Drapkina et al., 2021). Studies have shown that the level of evidence for off-label pediatric medications is low, and only 14% of off-label medications were assessed in high-quality studies. Furthermore, 37% of off-label medications are supported by no clinical studies (van der Zanden et al., 2022). Drug treatment available to pediatric patients will be markedly limited when a high level of evidence becomes necessary for off-label drug use.

This study presents a unique contribution as the first investigating off-label drug prescription patterns for pediatrics visiting the Otorhinolaryngology, Head, and Neck Surgery Department in China. Our findings are strengthened by a large dataset (22,487 prescriptions for 20,757 patients), providing robust estimates of off-label drug prescription and supporting stable regression analysis. This study, however, has also limitations. First, in this single-center study, we retrospectively collected data and only included outpatient medications of pediatrics undergoing otolaryngology, head, and neck surgery. Considering the differences between hospitals and regions, it is necessary to expand the sample size and include medical institutions in different regions for further research and validation. However, based on the size of our hospital and the comprehensive diagnosis and treatment strength of the Otolaryngology, Head, and Neck Surgery Departments in China, we believe that our study results are representative and valuable for future clinical practice. Second, this study provides an overview of the current status of off-label drug use and the associated factors. However, it was not within the scope of this study to determine whether off-label drug use is beneficial or harmful.

## 5 Conclusion

This study identified 22,487 off-label prescriptions, including 2,269 off-label prescriptions for pediatric patients in the Department of Otolaryngology, Head, and Neck Surgery. The incidence rate of off-label drug use was 10.09%. Patient’s age, number of prescribed drugs, and physician’s professional title were the factors affecting off-label drug use. Older pediatric patients received a greater variety of medications, increasing the likelihood of off-label drug use. Additionally, residents are more often prescribed off-label drugs compared to attending physicians, associate chief physicians, and chief physicians.

Based on these findings, future studies should systematically monitor the clinical outcomes of patients receiving off-label drugs. Furthermore, we aimed to provide evidence-based guidance for the use of off-label drugs by rigorously assessing their efficacy and safety.

## Data Availability

The original contributions presented in the study are included in the article/supplementary material, further inquiries can be directed to the corresponding author.
